# Actigraphy-derived physical activity levels and circadian rhythm parameters in patients with psoriatic arthritis: relationship with disease activity, mood, age and BMI

**DOI:** 10.1177/1759720X231174989

**Published:** 2023-07-04

**Authors:** Dylan McGagh, Niall McGowan, Chris Hinds, Kate E. A. Saunders, Laura C. Coates

**Affiliations:** Nuffield Department of Orthopaedics, Rheumatology and Musculoskeletal Sciences, University of Oxford, The Botnar Research Centre, Old Road, Headington, Oxford, OX3 7LD, UK; Sleep and Circadian Neuroscience Institute, Nuffield Department of Clinical Neurosciences, University of Oxford, Oxford, Oxfordshire, UK; Department of Psychiatry, University of Oxford, Oxford, UK; Oxford Digital Phenotyping Laboratory, Big Data Institute, University of Oxford, Oxford, UK; Department of Psychiatry, University of Oxford, Oxford, UK; Oxford Health NHS Foundation Trust, Warneford Hospital, Oxford, UK; Nuffield Department of Orthopaedics, Rheumatology and Musculoskeletal Sciences, University of Oxford, Oxford, UK

**Keywords:** circadian rhythm, epidemiology, mobile health, physical activity, psoriatic arthritis

## Abstract

**Background:**

Psoriatic arthritis (PsA) is associated with sleep disturbance, depression and a lifetime risk of obesity and cardiovascular disease. To date, there have been no studies investigating the relationship between objectively-measured physical activity (PA) levels and circadian rhythm disturbance with disease activity, daily symptoms and mood in patients with PsA.

**Objective:**

This pilot study aimed to investigate the relationship between disease activity, daily symptoms and mood on PA and circadian rhythm in PsA.

**Design:**

A prospective cohort study recruiting adults with PsA from rheumatology clinics at a single centre in the UK.

**Methods:**

Participants wore an actigraph and recorded their symptoms and mood on a daily basis via a smartphone app for 28 days. Time spent in sedentary, light and moderate-to-vigorous physical activity (MVPA) and parameters reflecting the circadian rhythm of the rest-activity pattern were derived. This included the onset time of the least active 5-h (L5) and most active 10-h (M10) daily consecutive periods and the relative amplitude (RA). The relationship factors between baseline clinical status, daily symptoms, PA and circadian measures were examined using linear mixed effect regression models.

**Results:**

Nineteen participants (8/19 female) were included. Participants with active PsA spent 63.87 min (95% CI: 18.5–109.3, *p* = 0.008) more in inactivity and 30.78 min (95% CI: 0.4–61.1, *p* = 0.047) less in MVPA per day compared to those in minimal disease activity (MDA). Age, body mass index and disease duration were also associated with PA duration. Participants with worse functional impairment had an M10 onset time 1.94 h (95% CI: 0.05–3.39, *p* = 0.011) later than those with no reported functional impairment. No differences were detected for L5 onset time or RA. Higher scores for positive mood components such as feeling energetic, cheerful and elated were associated with less time in inactivity and greater time spent in MVPA overall.

**Conclusion:**

Our study highlights differences in PA and circadian rest-activity pattern timing based on disease activity, disability and daily mood in PsA. Reduced PA levels in patients with active disease may contribute to the observed increased risk of cardiovascular and metabolic sequelae, with further studies exploring this need.

## Introduction

Psoriatic arthritis (PsA) is a chronic inflammatory disease which occurs in approximately 30% of patients with psoriasis. The disease can manifest as peripheral arthritis, axial involvement, enthesitis and dactylitis, and it leads to an increased lifetime risk of metabolic syndrome and cardiovascular disease (CVD).^1^ It is understood that underlying endothelial dysfunction secondary to chronic inflammation and lifestyle factors such as increased smoking rates and obesity contribute to the increased CVD risk in PsA.^2,3^ Due to the overall manifestations of PsA with inflammation affecting joints and tendons leading to pain, immobility and joint destruction; physical activity (PA) levels are expected to be lower than matched healthy controls.

A systematic review investigating PA in spondyloarthritis utilizing both objective actigraphic- and subjective self-reported measures found an inverse relationship between disease activity and level of physical activity with those in remission and low disease activity having higher PA levels.^4^ The benefits of exercise on pain, fatigue, functional status and disease activity have been previously demonstrated in two randomized controlled trials in PsA,^5,6^ with various treatment guidelines having been updated to integrate exercise recommendations into clinical practice.^7^

There have also been several studies investigating the prevalence and burden of sleep disturbance in psoriasis and PsA. The pooled prevalence of poor sleepers, assessed using the Pittsburgh Sleep Quality Index, was 84% (95% CI 78–90%, *I*^2^ = 0%).^8,9^ Sleep disturbance was also found to be significantly associated with greater anxiety, generalized pain, enthesitis, levels of C-reactive protein and lower quality of life.^8^ Haugeberg *et al.*^10^ demonstrated an association between pain, fatigue and worse functional impairment and sleep disturbance in PsA. These studies, whilst cross-sectional and relying on self-reported assessment of sleep impairment, suggest an important role between poor sleep and disease burden and activity in PsA.

Sleep and PA are regulated by homeostatic and circadian processes.^11^ Circadian rhythms are self-sustaining 24 hour rhythmic oscillations in a host of physiological parameters that contribute to the regulation of rest and activity.^12^ There is mounting evidence to suggest a role of the circadian clock in the pathogenesis and clinical presentation of inflammatory arthritis (IA).^13^ Symptoms of IA follow a circadian rhythm with joint stiffness, pain and swelling occurring in the morning time. This chronobiological presentation of symptoms in RA has been partly attributed to the underlying circadian rhythm patterns of cytokines and hormones.^14,15^ The rhythmic changes of symptoms in RA patients have been shown to be accompanied by circadian oscillations in pro-inflammatory cytokine concentrations, with the levels of key inflammatory cytokines, including interleukin-6 (IL-6), tumour necrosis factor-α (TNF-α) and interleukin-1β (IL-1β) found to be significantly elevated at night.^15,16^ Furthermore, there have been two recent studies in the general population investigating the relationship between blunted rest-activity cycles and immune signature which demonstrated a pro-inflammatory phenotype associated with circadian misalignment.^17,18^ There have been several population-level studies linking rotating shift work with the development of RA and psoriasis.^19–21^ Collectively, these laboratory and clinical studies suggest a role for disturbed sleep-wake cycles and circadian misalignment in the pathogenesis of these autoimmune conditions. There are yet to be any detailed studies into circadian misalignment in PsA; however, the shared overlying mechanisms with other IA and psoriasis suggest a mechanistic association.

Digital health approaches through objectively quantifying PA levels via actigraphy and assessing disease burden at increased frequency using smartphone applications present an opportunity for a more granular assessment of PsA. The marked heterogeneity observed across PsA presents a challenge for quantifying treatment responses in clinical practice and research trials which relies heavily on self-report methods. To date, there have been no studies investigating these digital approaches in PsA. This study aims to investigate the relationship between disease activity, daily symptoms and mood on PA and circadian rhythm in PsA.

## Methods

### Study design and setting

In this prospective cohort study, we used data from accelerometers and daily electronic questionnaires eliciting mood and disease symptoms to investigate the relationship between daily PA levels, circadian rest-activity patterns and fluctuations in self-reported mood and symptoms against baseline participant characteristics. Consecutive patients with PsA meeting inclusion criteria were approached from rheumatology clinics at a single centre in the UK.

### Sample size

As this was a feasibility study, no formal sample size calculation was performed. A target of 30 participants recruited over an 18-month period was set. This was modified to 25 participants to account for difficulties in recruitment due to the impact of COVID-19 pandemic on in-person clinic attendance.

### Eligibility

Participants aged ⩾18 and <80 years with a diagnosis of PsA [defined by the ClASsification criteria for Psoriatic ARthritis (CASPAR definition)] were included. Participants were excluded if they had any comorbid diagnoses associated with disturbed sleep, as determined by a consultant rheumatologist following interrogation of medical records and clinical history. Individuals were also excluded if their work included shift patterns or requirements to travel across time zones.

### Clinical and self-report assessment

Baseline disease activity was assessed using the minimal disease activity (MDA) criteria. Disease burden was measured using the Psoriatic Arthritis Impact of Disease-12 questionnaire. Functional impairment was measured using the Health Assessment Questionnaire (HAQ). The Patient Health Questionnaire-2 (PHQ-2) was used to screen for prevalent depression amongst our cohort.

### Measurement of PA and rest-activity patterns

PA and rest-activity patterns were objectively assessed using a tri-axial wrist accelerometer (GENEActiv^®^, Activinsights Ltd), which was worn continuously on the participants’ non-dominant wrist for 28 days. The accelerometers measure movement expressed as acceleration units at a 25 Hz measurement frequency. This measurement frequency was selected to facilitate data storage and battery life for an uninterrupted 28-day data collection.

Following the collection period, raw accelerometer data were extracted using the manufacturer software and processed using the open-source GGIR R-package (V2.8-0).^22^ GGIR was also used to detect abnormal values and non-wear time. Subsequently, the Euclidean Norm Minus One was determined from the signal vector magnitude by subtracting one gravitational unit from the three raw acceleration signals at each time stamp of collection. Only participants with at least 7 days of valid accelerometer data were included. Wear-time cut-off in order to include a day in the analysis was set to ⩾21 h. Duration of inactivity, light PA and moderate-to-vigorous physical activity (MVPA) were computed using previously validated thresholds ^23^: inactivity <30 mg; 30 mg ⩾ light PA < 100 mg; 100 mg ⩾ moderate PA < 400 mg; vigorous PA ⩾ 400 mg. PA levels were expressed in minutes per day and were calculated based on waking hours only; therefore, periods of inactivity do not capture periods where participants were asleep.

Non-parametric circadian rhythm analysis was performed to determine the amplitude of the circadian rest-activity rhythms and to examine the average phase and activity levels during the 5 h period of lowest activity (L5) and 10 h period of greatest activity (M10).^24^ The method for deriving these variables from GENEActiv accelerometers has been described in detail elsewhere.^25^

### Measurement of daily mood and disease symptoms

We utilized the Mezurio platform, a smartphone application developed at the University of Oxford to nest our daily questionnaires. Mezurio is an encrypted app which has been deployed in clinical studies and daily clinical practice to collect electronic patient-reported outcome measures (ePROMs) and cognitive exercises.^26,27^ We utilized the Mezurio platform to prompt participants to record their daily symptom burden by nesting a modified PsAID-9 questionnaire within the app. This recorded symptom severity for the preceding 24 hours across all domains on a scale of 0–10. Daily mood was measured using the validated Mood Zoom (MZ) questionnaire. MZ was initially developed to identify predominant mood states in longitudinal mood profiling in people with bipolar disorder and borderline personality disorder.^28^ We utilized a modified MZ questionnaire which expanded on the initial six descriptor items of (1) anxious, (2) elated, (3) sad, (4) angry, (5) irritable and (6) energetic to include (7) cheerful, (8) guilt, (9) shame and (10) doubt as these were deemed to be more generalizable to a wider population. Participants collected 28 days of questionnaires using the Mezurio app.

### Statistical analysis

Descriptive statistics were used for reporting baseline disease characteristics. Univariate linear mixed-effects regression (LMER) models were then deployed to investigate the relationship between sedentary time, light exercise and MVPA and baseline characteristics. We then built multivariate LMER models including age, disease duration, body mass index (BMI) and weekday type (weekend *versus* weekday) as fixed effects related to PA. We created a random-effects model using participant identification number as a random intercept. In order to investigate the relationship between daily mood and symptom fluctuations, we created LMER models regressing the PA measures against the matched day MZ and PsAID-9 recordings. Models investigating app data and PA controlled for age and BMI. Individual models were built against each mood component and matched activity levels resulting in 10 models. We adjusted the α-level to 0.005 to correct for multiple comparisons within these models. All mixed-effects models were fitted using maximum likelihood estimation. Satterthwaite’s method was used to generate sample statistics on the model estimates.^29^ All statistical analyses were conducted using RStudio (R version 4.2.1, RStudio Inc., Boston, MA) with the lme4 package (version 1.1-30) used for building linear mixed-effects models.^30^

### Ethics and reporting standards

The study conformed to the Declaration of Helsinki and was approved by local research ethics committee (REC 19/LO/1193). All participants gave their written informed consent. The reporting of this study conforms to the STROBE statement.^31^

## Results

### Participants

Twenty-five participants were enrolled and six patients were excluded either because they were lost to follow-up (*n* = 3), failed to complete sufficient accelerometer recordings (*n* = 2) or in one participant’s case their work pattern switched to include night shifts. The characteristics of the 19 participants included are presented in Table 1. Details on PsA treatments are presented in the Supplemental Materials.

**Table 1.** Participant characteristics at baseline.

Variables	PsA (*n* = 19)
Demographics	
Age (year)	52 ± 11
Female (%)	42.1
BMI	29.6 ± 9.0
Disease duration (year)	2 ± 1*
MDA achieved (%)	47.4
Comorbid psoriasis (%)	68.4
Peripheral-PsA subtype (%)	84.2
Axial-PsA subtype (%)	15.8
Global assessment	
Patient global activity (0–10)	3.1 ± 2.5
Patient-reported outcome	
HAQ	0.46 ± 0.38
Pain VAS (0–10)	3.3 ± 2.8
PsAID-9 score (0–10)	2.9 ± 1.9
PHQ-2 depression (%)	0

* Median and interquartile range.

BMI, body mass index; MDA, minimal disease activity; HAQ, Health Assessment Questionnaire; VAS, visual analogue scale; PsAID-9, Psoriatic Arthritis Impact of Disease-9; PHQ-2, Patient Health Questionnaire; PsA, psoriatic arthritis.

### Assessing PA with wrist-worn actigraphy is feasible in patients with PsA

A total of 342 eligible person-days (64.3% of the 532 days of paired actigraphy and daily app data available) were taken. Wear-time compliance with a paired app completion for those included in the final analysis was 17.2 ± 4.6 days. The number of valid week days was 12.3 ± 3 and valid weekend days was 4.9 ± 1.5. The mean time spent in inactivity was 602 ± 77 min per day; in light PA was 237 ± 68 min per day; and in MVPA was 90.5 ± 40.8 min.

### Impact of disease activity on PA levels in PsA

LMER models controlling for age, BMI, disease duration and weekday type (weekend *versus* weekday) identified a statistically significant association between MDA and levels of PA (Table 2). Participants with active PsA spent 63.87 min (95% CI: 18.5–109.3, *p* = 0.008) more in inactivity and 30.78 min (95% CI: 0.4–61.1, *p* = 0.047) less in MVPA per day compared to those in MDA. Age, BMI and disease duration were also associated with PA duration (Table 2) within the multivariate models. A 1-year increase in age was associated with 2.22 minutes (95% CI: 0.12–4.3, *p* = 0.04) more in inactivity and 1.18 minutes (95% CI: 0.45–2.81, *p* = 0.014) less in MVPA. There was no significant difference in activity levels between a weekday and weekend day.

**Table 2.** LMER model outputs investigating relationship between disease activity, BMI, age, disease duration and weekend effect on PA levels.

Predictors	Inactivity (mins/day)			Light PA (mins/day)			MVPA (mins/day)		
	Estimates	CI	*p*	Estimates	CI	*p*	Estimates	CI	*p*
(Intercept)	401.99	280.99 to 522.98	<**0.001**	303.51	151.22 to 455.80	**0.001**	207.38	126.06 to 288.69	<**0.001**
MDA	−63.87	−109.23 to −18.52	**0.008**	34	−22.80 to 90.80	0.225	30.78	0.43 to 61.13	**0.047**
Age	2.22	0.12 to 4.33	**0.04**	−0.78	−3.42 to −1.87	0.546	−1.83	−3.24 to −0.42	**0.014**
BMI	2.99	0.54 to 5.44	**0.019**	−0.58	−3.63 to −2.48	0.697	−1.18	−2.81 to 0.45	0.147
Disease duration	0.63	0.26 to 1.00	**0.002**	−0.42	−0.88 to −0.04	0.074	0.04	−0.20 to 0.29	0.706
Weekend	−8.65	−34.11 to 16.81	0.504	−20.84	−33.49 to −8.20	**0.001**	−7.44	−15.81 to 0.92	0.081

BMI, body mass index; LMER, linear mixed-effects regression; MDA, minimal disease activity; MVPA, moderate-to-vigorous physical activity; PA, physical activity. P-value < 0.05.

### Impact of disease activity and disability on circadian rhythm measures

LMER models investigating the relationship between disease activity and the circadian rhythm of the rest-activity cycle found a non-significant trend between achieving MDA and an earlier M10 onset time (Supplemental Table S1). LMER models further investigating the individual components comprising MDA criteria against L5 and M10 onset times identified a significant association between delayed M10 onset time and worse functional impairment, as measured using the HAQ (Supplemental Table S2). A full model investigating the relationship between HAQ and M10 controlling for age, BMI, disease duration and weekend effect demonstrated that a 1-point increase in HAQ score was associated with a 1.94 h (95% CI: 0.49–3.39, *p* = 0.011) delayed M10 onset time (Table 3). Achieving MDA or HAQ was not associated with a difference in L5 onset time. This suggests, within these models, that disease activity or disability is not associated with a night-time phase delay in the circadian rhythm in patients with PsA.

**Table 3.** LMER model outputs investigating relationship between HAQ, BMI, age, disease duration and weekend effect on circadian rhythm measures.

Predictors	L5 Time			M10 Time			RA		
	Estimates	CI	*p*	Estimates	CI	*p*	Estimates	CI	*p*
(Intercept)	24.16	21.33 to 26.98	<**0.001**	9.43	6.52 to 12.34	<**0.001**	1.01	0.80 to 1.23	<**0.001**
HAQ	1.3	−0.10 to 2.71	0.067	1.94	0.49 to 3.39	**0.011**	−0.05	−0.16 to 0.05	0.299
Age	−0.01	−0.06 to 0.03	0.564	−0.02	−0.07 to 0.03	0.477	0	−0.01 to −0.00	**0.046**
BMI	0.02	−0.04 to 0.08	0.536	−0.01	−0.07 to 0.05	0.735	0	−0.00 to 0.00	0.826
Disease duration	0.01	0.00 to 0.02	**0.029**	0	−0.01 to 0.01	0.518	0	−0.00 to 0.00	0.745
Weekend	0.46	−0.03 to 0.95	0.065	0.27	−0.16 to 0.70	0.223	0	−0.02 to 0.01	0.71

BMI, body mass index; HAQ, Health Assessment Questionnaire; LMER, linear mixed-effects regression; RA, relative amplitude. P-value < 0.05.

### Relationship between PA and daily app questionnaires

Models investigating the relationship between daily mood and PA levels for the matched day identified a clear bi-directional association between feeling energetic, cheerful and elated on reduced inactivity and increased MVPA, as shown in Table 4. Negatively grouped mood components such as feeling sad, ashamed, irritable or angry had no significant relationship with PA levels (Supplemental Table S3). LMER models investigating the relationship between daily PsA symptoms and matched day PA levels failed to identify any relationship (Supplemental Table S2). Notably, total PsAID-9 score, pain or fatigue levels were not significantly associated with any PA levels for the matched day (Supplemental Table S4).

**Table 4.** LMER model outputs investigating relationship between daily mood on PA level.

Predictors	Inactivity (mins/day)			Light PA (mins/day)			MVPA (mins/day)		
	Estimates	CI	*p*	Estimates	CI	*p*	Estimates	CI	*p*
(Intercept)	425.86	271.26 to 580.46	<0.001	304.15	150.79 to 457.50	<0.001	180.59	109.61 to 251.58	<0.001
Energetic	−41.39	−60.21 to −22.58	<0.001	14.91	4.16 to 25.65	0.007*	13.3	6.56 to 20.04	<0.001
(Intercept)	438.59	294.70 to 582.47	<0.001	301.83	147.98 to 455.68	<0.001	171.52	98.95 to 244.09	<0.001
Cheerful	−37.43	−55.06 to −19.80	<0.001	12.48	2.40 to 22.55	0.015*	14.22	7.91 to 20.53	<0.001
(Intercept)	427.27	287.10 to 567.45	<0.001	307.59	153.62 to 461.57	<0.001	179.41	106.88 to 251.93	<0.001
Elated	−38.88	−58.22 to −19.54	<0.001	11.8	0.28 to 23.32	0.045*	12.81	5.61 to 20.00	0.001

All models control for age and BMI. All significant findings were significant within univariate models with age and BMI removed; accepted α-level of 0.005 to correct for multiple models and spurious statistical significance. All items in bold represent statistical significance.

* Deemed not significant post-Bonferroni correction.

MVPA, moderate-to-vigorous physical activity; PA, physical activity.

## Discussion

Our study demonstrates a robust association between increased disease activity and reduced PA in patients with PsA. These findings open a potential secondary causal path to explain the increased lifetime risk of CVD and CVD-related morbidity in patients with PsA.^1^ There has been one previous actigraphy study exploring PA in PsA which demonstrated comparable PA levels between PsA and healthy controls with a secondary analysis showing reduced PA in patients with increased disease activity, as measured with the DAPSA score which is further supported by the current findings.^32^ Mechanistically, the increased risk of CVD in PsA has been explained by chronic low-level inflammation leading to endothelial dysfunction and dyslipidaemia.^1,2^ The findings from this cohort study would support further longitudinal research investigating level and type of exercise on incidence of CVD, factoring in the impact of disease severity.

Our study has also demonstrated disruption to circadian rest-activity patterns in the form of delayed M10 onset time related to functional impairment in PsA. Delayed M10 onset time as a marker of delayed activity onset may represent morning stiffness, pain and immobility associated with increased disease activity and is worth further investigation as an indication of early morning symptoms. Work disability has previously been shown to be associated with worse HAQ scores in PsA.^33,34^ The findings of delayed M10 time associated with worse functional impairment and disability, as measured with the HAQ, may represent differences in employment status across our cohort or may capture those with more advanced disease with irreversible joint damage. The HAQ has been shown to be a sensitive marker to treatment response in clinical trials,^35,36^ thus highlighting the potential for M10 onset time as a novel and objectively estimated contemporaneous marker of treatment response. It would also be of utility to collect blood samples and correlate markers of inflammation with circadian rest-activity patterns in patients with PsA. Recent work has shown an association between circadian misalignment and a pro-inflammatory immune signature and further exploring these findings in an IA population is of importance.^17,18^

Our data also demonstrate the feasibility of collecting paired daily symptom app and actigraphy data in patients with PsA and to our knowledge is the first such study deploying this approach in this patient cohort. The findings demonstrating the impact of daily mood fluctuations on the level of PA also highlight the importance of considering factors beyond disease activity on PA levels. The current study was not powered to investigate the influence of comorbid mood or anxiety disorders on PA levels, as demonstrated by the lack of participants that screened positive for depression on PHQ-2 at baseline. There is evidence to suggest that comorbid depression and anxiety in PsA leads to a lower likelihood of achieving remission, with recent research showing that depression and mood disorders can predominantly influence patient-reported disease domains.^37,38^ The relationship between baseline and daily mood and PA, circadian rhythm and disease activity in PsA could be explored in larger cohorts utilizing the approaches from the current study. Our study failed to identify a difference in PA levels related to daily fluctuations in disease symptoms, as measured through the daily PsAID-9 questionnaire. This could likely be explained by a lack of within-participant variation in scoring across the study collection with 28 days a relatively short time to experience change from baseline PsAID-9. An interesting approach worth exploring would be to recreate work done in rheumatoid arthritis and idiopathic inflammatory myopathy patient cohorts where longitudinally collected app data on weekly self-reported flares and daily disease symptoms across a 3-month collection period permitted investigation into the profile of symptoms preceding disease flare.^39,40^

### Limitations

There are a number of potential limitations to consider with the findings from this study. Importantly, there has been some evidence to suggest that participants recruited to digital health studies tend to be younger and have lower overall disease activity.^41^ Whilst we made efforts to recruit across the spectrum of participants who presented to routine clinic, we have not formally factored this into our overall recruitment and may have introduced a potential selection bias for participants with higher digital literacy and lower overall disease activity. Another limitation of this study is related to the development of the daily symptom and mood-based questionnaires. This study could have benefited from increased patient involvement in the design of these digital measurement tools to ensure validity to this specific patient population. We have utilized the Mezurio platform which has been extensively piloted and validated in over 80,000 participants so is optimized for participant usage but more for a general population. A final limitation of our study is the impact of the COVID-19 pandemic on recruitment numbers. The shift to increased number of telemedicine appointments in place of routine face-to-face clinic attendance made it more difficult to recruit patients to the study.

## Conclusions

Collectively, these data demonstrate the potential utility of digital phenotyping of mood and daily disease symptoms within PsA cohorts. Remote daily monitoring could have applications in PsA clinical trials to aid efficacy assessment.

## Supplemental Material

sj-docx-1-tab-10.1177_1759720X231174989 – Supplemental material for Actigraphy-derived physical activity levels and circadian rhythm parameters in patients with psoriatic arthritis: relationship with disease activity, mood, age and BMI Click here for additional data file. Supplemental material, sj-docx-1-tab-10.1177_1759720X231174989 for Actigraphy-derived physical activity levels and circadian rhythm parameters in patients with psoriatic arthritis: relationship with disease activity, mood, age and BMI by Dylan McGagh, Niall McGowan, Chris Hinds, Kate E. A. Saunders and Laura C. Coates in Therapeutic Advances in Musculoskeletal Disease

## Declarations

**Ethics approval and consent to participate:** The study conformed to the Declaration of Helsinki and was approved by the local research ethics committee (REC 19/LO/1193).

**Consent for publication:** All participants gave their written informed consent to participate and for any findings to be published.

**Third-party submissions:** Not applicable.

**Availability of data and materials:** Data can be made available at the request of the corresponding author.
