# Supplementary material for: Actigraphy-derived physical activity levels and circadian rhythm parameters in patients with psoriatic arthritis: relationship with disease activity, mood, age and BMI
Source: Ther Adv Musculoskelet Dis. 2023 Jul 4;15:1759720X231174989. doi: 10.1177/1759720X231174989 (PMC10331082; doi:10.1177/1759720X231174989)
Supplement: sj-docx-1-tab-10.1177_1759720X231174989 – Supplemental material for Actigraphy-derived physical activity levels and circadian rhythm parameters in patients with psoriatic arthritis: relationship with disease activity, mood, age and BMI [file sj-docx-1-tab-10.1177_1759720X231174989.docx]

# Supplementary Materials

## Methods

### Mood Zoom Study Schedule

### Mezurio based questionnaires

**Questionnaire Title: PsAID**

Questionnaire Subtitle: Please rate how much you have been bothered by each of the given problems over the past day.

Title: Pain

Subtitle: Select the number that best describes the pain you felt due to your psoriatic arthritis during the last day

-0 1 2 3 4 5 6 7 8 9 10

None Extreme

Title: Fatigue

Subtitle: Select the number that best describes the overall level of fatigue due to your psoriatic arthritis you have experienced during the last day

Choices:

-0 1 2 3 4 5 6 7 8 9 10

No fatigue Totally exhausted

Title: Skin problems

Subtitle: Select the number that best describes the skin problems including itching you felt due to your psoriatic arthritis during the last day

Choices:

-0 1 2 3 4 5 6 7 8 9 10

None Extreme

Title: Work and/or leisure activities

Subtitle: Select the number that best describes the difficulties you had to participate fully in work and/or leisure activities due to your psoriatic arthritis during the last day:

Choices:

-0 1 2 3 4 5 6 7 8 9 10

None Extreme

Title: Functional capacity

Subtitle: Select the number that best describes the difficulty you had in doing daily physical activities due to your psoriatic arthritis during the last day:

Choices:

-0 1 2 3 4 5 6 7 8 9 10

No difficulty Extreme difficulty

Title: Discomfort

Subtitle: Select the number that best describes the feeling of discomfort and annoyance with everyday tasks due to your psoriatic arthritis during the last day

Choices:

-0 1 2 3 4 5 6 7 8 9 10

None Extreme

Title: Sleep disturbance

Subtitle: Select the number that best describes the sleep difficulties (i.e., resting at night) you felt due to your psoriatic arthritis during the last day

Choices:

-0 1 2 3 4 5 6 7 8 9 10

No difficulty Extreme difficulty

Title: Coping

Subtitle: Considering your psoriatic arthritis overall, how well did you cope (manage, deal, make do) with your psoriatic arthritis during the last day?

Choices:

-0 1 2 3 4 5 6 7 8 9 10

Very well Very poorly

Title: Anxiety

Subtitle: Select the number that best describes the level of anxiety, fear and uncertainty due to your psoriatic arthritis you have experienced during the last day:

Choices:

-0 1 2 3 4 5 6 7 8 9 10

None Extreme

Title: Thank you

Subtitle: You have successfully completed your PsAID questionnaire

**Questionnaire Title: Mood Zoom**

Questionnaire Subtitle: Please rate to what extent the following words represent how you currently feel:

Title: Anxious

Subtitle: Please rate your current mood

Choices:

- Not at all

- Slightly

- Moderately

- Very

- Extremely

Title: Elated

Subtitle: Please rate your current mood

Choices:

- Not at all

- Slightly

- Moderately

- Very

- Extremely

Title: Sad

Subtitle: Please rate your current mood

Choices:

- Not at all

- Slightly

- Moderately

- Very

- Extremely

Title: Angry

Subtitle: Please rate your current mood

Choices:

- Not at all

- Slightly

- Moderately

- Very

- Extremely

Title: Irritable

Subtitle: Please rate your current mood

Choices:

- Not at all

- Slightly

- Moderately

- Very

- Extremely

Title: Energetic

Subtitle: Please rate your current mood

Choices:

- Not at all

- Slightly

- Moderately

- Very

- Extremely

Title: Cheerful

Subtitle: Please rate your current mood

Choices:

- Not at all

- Slightly

- Moderately

- Very

- Extremely

Title: Guilt

Subtitle: Please rate your current feelings

Choices:

- Not at all

- Slightly

- Moderately

- Very

- Extremely

Title: Shame

Subtitle: Please rate your current feelings

Choices:

- Not at all

- Slightly

- Moderately

- Very

- Extremely

Title: Self-doubt

Subtitle: Please rate your current feelings

Choices:

- Not at all

- Slightly

- Moderately

- Very

- Extremely

Title: Thanks

Subtitle: Thank you for completing this session's Mood Zoom rating.

## Results

Supplementary table 1. LMER model outputs investigating relationship between disease activity, BMI, age, disease duration and weekend effect on circadian rhythm measures.

|  | **L5 Onset Time** | | | **M10 Onset Time** | | |
| --- | --- | --- | --- | --- | --- | --- |
| *Predictors* | *Estimates* | *CI* | *p* | *Estimates* | *CI* | *p* |
| (Intercept) | 24.15 | 21.05 – 27.25 | **<0.001** | 9.52 | 6.37 – 12.67 | **<0.001** |
| MDA | -0.16 | -1.32 – 1.00 | 0.779 | -1.13 | -2.31 – 0.05 | 0.061 |
| Age | -0.01 | -0.06 – 0.04 | 0.717 | 0 | -0.06 – 0.05 | 0.892 |
| BMI | 0.04 | -0.03 – 0.10 | 0.255 | 0.01 | -0.05 – 0.07 | 0.734 |
| Disease duration | 0.01 | -0.00 – 0.02 | 0.05 | 0 | -0.01 – 0.01 | 0.472 |
| Weekend | 0.46 | -0.03 – 0.95 | 0.064 | 0.27 | -0.16 – 0.70 | 0.216 |

Supplementary table 2. LMER models investigating the relationship between component of MDA on L5 and M10 onset times.

|  | **L5 Onset Time** | | | **M10 Onset Time** | | |
| --- | --- | --- | --- | --- | --- | --- |
| *Predictors* | *Estimates* | *CI* | *p* | *Estimates* | *CI* | *p* |
| (Intercept) | 25.33 | 24.03 – 26.63 | **<0.001** | 9.57 | 8.59 – 10.56 | **<0.001** |
| HAQ | 1.57 | -0.35 – 3.49 | 0.103 | 2.03 | 0.58 – 3.48 | **0.009** |
| Psoriasis | -0.7 | -2.09 – 0.69 | 0.301 | -0.94 | -1.99 – 0.12 | 0.078 |
| Pain VAS | -0.01 | -0.03 – 0.01 | 0.296 | -0.02 | -0.04 – -0.00 | **0.02** |
| Global assessment | 0 | -0.04 – 0.04 | 0.99 | 0 | -0.03 – 0.03 | 0.783 |
| TJC | 0.06 | -0.12 – 0.24 | 0.519 | 0.07 | -0.07 – 0.20 | 0.308 |
| SJC | -0.19 | -0.43 – 0.05 | 0.109 | -0.19 | -0.37 – -0.01 | **0.04** |
| Linear-mixed effects model investigating the individual components of Minimal Disease Activity (MDA) criteria on L5 and M10 onset times; HAQ, Health Assessment Questionnaire; VAS, visual analogue scale; TJC, tender joint count; SJC, swollen joint count. | | | | | | |

Supplementary table 3. Impact of negatively grouped mood domains on PA levels

|  |  | **Inactivity (mins/day)** | | | **Light PA (mins/day)** | | | **MVPA (mins/day)** | | |
| --- | --- | --- | --- | --- | --- | --- | --- | --- | --- | --- |
|  | *Predictors* | *Estimates* | *CI* | *p* | *Estimates* | *CI* | *p* | *Estimates* | *CI* | *p* |
| *Negative mood* |  |  |  |  |  |  |  |  |  |  |
|  | (Intercept) | 300.92 | 141.81 – 460.02 | <0.001 | 348.83 | 189.01 – 508.64 | <0.001 | 215.66 | 131.28 – 300.04 | <0.001 |
|  | Sad | 32.04 | 1.49 – 62.59 | 0.04* | -11.68 | -28.68 – 5.33 | 0.178 | -7.6 | -18.53 – 3.34 | 0.173 |
|  | (Intercept) | 371.3 | 197.56 – 545.04 | <0.001 | 350.28 | 189.18 – 511.37 | <0.001 | 224.09 | 138.19 – 309.98 | <0.001 |
|  | Guilt | -11.36 | -53.05 – 30.32 | 0.592 | -12.57 | -36.89 – 11.74 | 0.31 | -12.78 | -28.16 – 2.59 | 0.103 |
|  | (Intercept) | 333.32 | 169.11 – 497.53 | <0.001 | 332.89 | 173.87 – 491.91 | <0.001 | 212.49 | 128.59 – 296.39 | <0.001 |
|  | Irritable | 11.02 | -14.07 – 36.11 | 0.388 | -1.72 | -15.51 – 12.07 | 0.807 | -5.16 | -14.02 – 3.70 | 0.253 |
|  | (Intercept) | 351.01 | 184.33 – 517.68 | <0.001 | 342.6 | 184.25 – 500.94 | <0.001 | 212.45 | 127.67 – 297.24 | <0.001 |
|  | Shame | 1.26 | -34.98 – 37.49 | 0.946 | -8.56 | -28.94 – 11.82 | 0.409 | -6.13 | -19.17 – 6.91 | 0.356 |
|  | (Intercept) | 294.14 | 128.91 – 459.36 | 0.001 | 310.21 | 149.62 – 470.81 | <0.001 | 206.74 | 119.12 – 294.37 | <0.001 |
|  | Doubt | 24.25 | -5.72 – 54.22 | 0.112 | 8.14 | -9.83 – 26.12 | 0.373 | -1.42 | -12.85 – 10.00 | 0.806 |
|  | (Intercept) | 306.38 | 141.84 – 470.91 | <0.001 | 338.09 | 175.47 – 500.72 | <0.001 | 216.28 | 130.85 – 301.70 | <0.001 |
|  | Anxious | 18.8 | -7.52 – 45.13 | 0.161 | -3.35 | -18.71 – 12.01 | 0.668 | -5.25 | -15.01 – 4.51 | 0.291 |
|  | (Intercept) | 329.67 | 166.10 – 493.25 | <0.001 | 355.45 | 188.97 – 521.93 | <0.001 | 217.54 | 132.37 – 302.71 | <0.001 |
|  | Angry | 16.7 | -17.54 – 50.94 | 0.338 | -18.48 | -37.16 – 0.21 | 0.053 | -10.27 | -22.33 – 1.79 | 0.095 |
|  | All models control for age and BMI. All significant findings were significant within univariate models with age and BMI removed; Accepted α-level of 0.005 to correct for multiple models and spurious statistical significance ; *deemed not significant post-Bonferroni correction; MVPA, moderate-to-vigorous physical activity. | | | | | | | | | |

Supplementary table 4. Impact of PsAID-9 total score, pain and fatigue on PA levels

|  | **Inactivity (mins/day)** | | | **Light PA (mins/day)** | | | **MVPA (mins/day)** | | |
| --- | --- | --- | --- | --- | --- | --- | --- | --- | --- |
| *Predictors* | *Estimates* | *CI* | *p* | *Estimates* | *CI* | *p* | *Estimates* | *CI* | *p* |
| (Intercept) | 372.62 | 205.73 – 539.52 | **<0.001** | 320.79 | 155.60 – 485.99 | **<0.001** | 212.35 | 124.57 – 300.13 | **<0.001** |
| PsAID-9 total | -6.09 | -24.28 – 12.11 | 0.511 | 2.73 | -14.43 – 19.89 | 0.754 | -2.73 | -12.06 – 6.60 | 0.565 |
| (Intercept) | 344.7 | 181.74 – 507.66 | **<0.001** | 331.2 | 173.74 – 488.65 | **<0.001** | 208.16 | 125.24 – 291.08 | **<0.001** |
| Pain | 2.66 | -7.18 – 12.50 | 0.596 | -0.43 | -6.22 – 5.37 | 0.885 | -1.53 | -5.19 – 2.13 | 0.413 |
| (Intercept) | 371.13 | 210.90 – 531.36 | **<0.001** | 318.05 | 160.78 – 475.31 | **<0.001** | 194.39 | 108.05 – 280.73 | **<0.001** |
| Fatigue | -4.6 | -12.00 – 2.79 | 0.222 | 2.93 | -0.99 – 6.84 | 0.143 | 2.2 | -0.34 – 4.74 | 0.089 |

Supplementary table 5a. Participants’ medications at baseline.

| csDMARDs |  |  |  |
| --- | --- | --- | --- |
|  | Methotrexate | Leflunomide | Sulfalazine |
|  | 14 | 3 | 2 |
|  |  |  |  |
| bDMARD |  |  |  |
|  | anti-TNFα | anti-IL12/23 | anti-IL17 |
|  | 6 | 1 | 2 |
| Key; csDMARD, conventional synthetic disease-modifying antirheumatic drug; bDMARD, biologic disease-modifying antirheumatic drug; TNFα, tumour necrosis factor-α | | | |

Suppplementary table 5b. Number of medications per participant at baseline.

| No agent | csDMARDs only | bDMARDs only | Combination |
| --- | --- | --- | --- |
| 10.5 (2/19) | 42.1 (8/19) | 5.3 (1/19) | 42.1 (8/19) |
|  |  |  |  |
| Key; csDMARD, conventional synthetic disease-modifying antirheumatic drug; bDMARD, biologic disease-modifying antirheumatic drug; TNFα, tumour necrosis factor-α | | | |

STROBE Statement—checklist of items that should be included in reports of observational studies

|  | **Item No.** | **Recommendation** | **Page  No.** | **Relevant text from manuscript** |
| --- | --- | --- | --- | --- |
| **Title and abstract** | 1 | (*a*) Indicate the study’s design with a commonly used term in the title or the abstract | 2 | Lines 39-41 |
|  |  | (*b*) Provide in the abstract an informative and balanced summary of what was done and what was found | 2 | Lines 31-63 |
| **Introduction** | | | |  |
| Background/rationale | 2 | Explain the scientific background and rationale for the investigation being reported | 5 | Lines 85-132 |
| Objectives | 3 | State specific objectives, including any prespecified hypotheses | 5 | Lines 134-140 |
| **Methods** | | | |  |
| Study design | 4 | Present key elements of study design early in the paper | 6 | Lines 150-210 |
| Setting | 5 | Describe the setting, locations, and relevant dates, including periods of recruitment, exposure, follow-up, and data collection | 6 | Lines 150-210 |
| Participants | 6 | (*a*) *Cohort study*—Give the eligibility criteria, and the sources and methods of selection of participants. Describe methods of follow-up  *Case-control study*—Give the eligibility criteria, and the sources and methods of case ascertainment and control selection. Give the rationale for the choice of cases and controls  *Cross-sectional study*—Give the eligibility criteria, and the sources and methods of selection of participants | 6 | Lines 161 - 167 |
|  |  | (*b*) *Cohort study*—For matched studies, give matching criteria and number of exposed and unexposed  *Case-control study*—For matched studies, give matching criteria and the number of controls per case | NA | NA |
| Variables | 7 | Clearly define all outcomes, exposures, predictors, potential confounders, and effect modifiers. Give diagnostic criteria, if applicable |  |  |
| Data sources/ measurement | 8* | For each variable of interest, give sources of data and details of methods of assessment (measurement). Describe comparability of assessment methods if there is more than one group | *Supplemental* |  |
| Bias | 9 | Describe any efforts to address potential sources of bias | 8 | Lines 212-226 |
| Study size | 10 | Explain how the study size was arrived at | 6 | Lines 157-160 |

Continued on next page

| Quantitative variables | 11 | Explain how quantitative variables were handled in the analyses. If applicable, describe which groupings were chosen and why | 6 | Lines 157-160 |
| --- | --- | --- | --- | --- |
| Statistical methods | 12 | (*a*) Describe all statistical methods, including those used to control for confounding | 6 | Lines 157-160 |
|  |  | (*b*) Describe any methods used to examine subgroups and interactions | NA | NA |
|  |  | (*c*) Explain how missing data were addressed | 9 | Lines 252-255 |
|  |  | (*d*) *Cohort study*—If applicable, explain how loss to follow-up was addressed  *Case-control study*—If applicable, explain how matching of cases and controls was addressed  *Cross-sectional study*—If applicable, describe analytical methods taking account of sampling strategy | NA | NA |
|  |  | (*e*) Describe any sensitivity analyses |  |  |
| **Results** | | | | |
| Participants | 13* | (a) Report numbers of individuals at each stage of study—eg numbers potentially eligible, examined for eligibility, confirmed eligible, included in the study, completing follow-up, and analysed | Included throughout |  |
|  |  | (b) Give reasons for non-participation at each stage | 6 | Lines 157-160 |
|  |  | (c) Consider use of a flow diagram | NA |  |
| Descriptive data | 14* | (a) Give characteristics of study participants (eg demographic, clinical, social) and information on exposures and potential confounders | 9 | Description of cohort in results: Lines 245-248 |
|  |  | (b) Indicate number of participants with missing data for each variable of interest | 9 | Lines 244-248 |
|  |  | (c) *Cohort study*—Summarise follow-up time (eg, average and total amount) | 9 | Lines 254 – 257: description of data collected |
| Outcome data | 15* | *Cohort study*—Report numbers of outcome events or summary measures over time | *NA* | *NA* |
|  |  | *Case-control study—*Report numbers in each exposure category, or summary measures of exposure | NA | *NA* |
|  |  | *Cross-sectional study—*Report numbers of outcome events or summary measures |  |  |
| Main results | 16 | (*a*) Give unadjusted estimates and, if applicable, confounder-adjusted estimates and their precision (eg, 95% confidence interval). Make clear which confounders were adjusted for and why they were included | Included in results throughout |  |
|  |  | (*b*) Report category boundaries when continuous variables were categorized |  |  |
|  |  | (*c*) If relevant, consider translating estimates of relative risk into absolute risk for a meaningful time period |  |  |

Continued on next page

| Other analyses | 17 | Report other analyses done—eg analyses of subgroups and interactions, and sensitivity analyses |  |  |
| --- | --- | --- | --- | --- |
| **Discussion** | | | | |
| Key results | 18 | Summarise key results with reference to study objectives | The following were discussed throughout. |  |
| Limitations | 19 | Discuss limitations of the study, taking into account sources of potential bias or imprecision. Discuss both direction and magnitude of any potential bias |  |  |
| Interpretation | 20 | Give a cautious overall interpretation of results considering objectives, limitations, multiplicity of analyses, results from similar studies, and other relevant evidence |  |  |
| Generalisability | 21 | Discuss the generalisability (external validity) of the study results |  |  |
| **Other information** | |  | | |
| Funding | 22 | Give the source of funding and the role of the funders for the present study and, if applicable, for the original study on which the present article is based |  |  |

*Give information separately for cases and controls in case-control studies and, if applicable, for exposed and unexposed groups in cohort and cross-sectional studies.

**Note:** An Explanation and Elaboration article discusses each checklist item and gives methodological background and published examples of transparent reporting. The STROBE checklist is best used in conjunction with this article (freely available on the Web sites of PLoS Medicine at http://www.plosmedicine.org/, Annals of Internal Medicine at http://www.annals.org/, and Epidemiology at http://www.epidem.com/). Information on the STROBE Initiative is available at www.strobe-statement.org.
